# A systematic review and bayesian meta-analysis of medical devices used in chronic pain management

**DOI:** 10.1038/s41598-024-63499-6

**Published:** 2024-06-12

**Authors:** Ashish Shetty, Gayathri Delanerolle, Chunli Deng, Anish Thillainathan, Heitor Cavalini, Xiaojie Yang, Yassine Bouchareb, Amy Boyd, Peter Phiri, Jian Qing Shi, Timothy Deer

**Affiliations:** 1 University College London Hospitals NHS Foundation Trust, London, UK; 2 University College London, 235, Euston Road, London, NW1 2BU UK; 3 Nuffield Department of Primary Care Health Sciences, University of Oxford, Oxford, OX3 7JX UK; 4 Southern University of Science and Technology, Shenzhen, 518055 China; 5 Psychology Department, Faculty of Environmental and Life Sciences, University of Southampton, Southampton, SO17 1BJ UK; 6 Southern Health NHS Foundation Trust, Southampton, SO40 2RZ UK; 7 School of Statistics and Mathematics, Yunnan University of Finance and Economics, Kunming, China; 8 National Centre for Applied Mathematics Shenzhen, Shenzhen, China; 9 Nuffield Department of Medicine, University of Oxford, Oxford, OX3 7JX UK; 10 Digital Evidence Based Medicine Lab, Oxford, UK; 11 College of Medicine and Health Sciences, Sultan Qaboos University, Muscat, Oman; 12 The Spine and Nerve Center of the Virginias, West Virginia University Hospitals, Charleston, WV USA

**Keywords:** Public health, Medical research, Neuropathic pain

## Abstract

Whilst^.^ pharmacological therapies remain the cornerstone of pain management in chronic pain, factors including the current opioid epidemic have led to non-pharmacological techniques becoming a more attractive proposition. We explored the prevalence of medical device use and their treatment efficacy in non-cancer pain management. A systematic methodology was developed, peer reviewed and published in PROSPERO (CRD42021235384). Key words of *medical device, pain management devices, chronic pain, lower back pain, back pain, leg pain* and *chronic pelvic pain* using Science direct, PubMed, Web of Science, PROSPERO, MEDLINE, EMBASE, PorQuest and ClinicalTrials.gov. All clinical trials, epidemiology and mixed methods studies that reported the use of medical devices for non-cancer chronic pain management published between the 1st of January 1990 and the 30th of April 2022 were included. 13 studies were included in systematic review, of these 6 were used in the meta-analysis. Our meta-analysis for pain reduction showed that transcutaneous electrical nerve stimulation combined with instrument-assisted soft tissue mobilization treatment and pulsed electromagnetic therapy produced significant treatment on chronic lower back pain patients. Pooled evidence revealed the use of medical device related interventions resulted in 0.7 degree of pain reduction under a 0–10 scale. Significant improvement in disability scores, with a 7.44 degree reduction in disability level compared to a placebo using a 50 score range was also seen. Our analysis has shown that the optimal use of medical devices in a sustainable manner requires further research, needing larger cohort studies, greater gender parity, in a more diverse range of geographical locations.

## Introduction

Chronic pain is a complex condition that is burdensome at an individual and societal level. It impacts approximately 20% of the global population with significant mobility restrictions, emotional distress, social isolation and financial difficulty^1,2^. The impact on society is significant, with health care expenses and lost productivity costing European economies over 200 billion dollars and the US economy 635 billion dollars each year^3^. This is reaffirmed by The Global Burden of Disease study 2016 which highlighted high prevalence of pain and pain-related comorbidities as a significant source of disability and disease burden globally^4,5^. Chronic pain populations are heterogeneous and this presents many challenges to patients, clinicians, clinical researchers and policy makers to design healthcare services that can meet the complex demands. Chronic pain prevalence and incidence varies by gender, biological sex and other social determinants. Epidemiological studies show older women, people from lower socioeconomic backgrounds and those with physical and psychological comorbidities are more likely to be at risk of long-term chronic pain^6^. An aging population means the risk of long-term chronic pain management is ever-increasing due to increased exposure to comorbidities^7^.

These statistics are further impacted by changes to the global migratory patterns between developed, emerging and developing countries. Lack of government policy, inadequate resources precluding the formation of chronic pain clinics and limited access to effective treatments lead to inadequate management of chronic pain in low-income countries^8–10^. Overcoming this disparity requires a focus on education of health care professionals, building research capacity, addressing cultural beliefs and stigmas related to pain and increasing availability of pharmacological therapies and medical devices. In high-income countries, migratory patterns change, making the tracking of changes in prevalence and incidence of those with chronic pain challenging^11–13^.

Pharmacological therapies have remained the cornerstone of pain management which influenced non-cancerous chronic pain. In particular, the current opioid epidemic indicates global consumption of pharmacological regimens doubling from 3.01 billion defined daily doses each year to 7.35 billion defined daily doses between 2001 and 2013^14,15^. Increases in opioid addiction and vulnerabilities to overdosing have led to a rise in global mortality, now at approximately 350,000 deaths per annum^16^. Despite this worrisome increase in morbidity and mortality related to pharmacological treatments, the overall benefit has been not shown to have significant efficacy, as based on the number needed to treat for most pain related medications evaluations.

Therefore, non-pharmacological techniques have become more attractive to all stakeholders. Non-pharmacological treatments for chronic pain can be categorised into two primary categories of medical devices and complex or combination treatments. For the purpose of this study, a medical device ‘can be any instrument, apparatus, implement, machine, appliance, implant, reagent for in vitro use, software, material or other similar or related article, intended by the manufacturer to be used, alone or in combination for a medical purpose^17^’. One of those device categories, termed neuromodulation devices, is based on changing the signal of the pain pathway from the peripheral nerve to the brain processing areas. The original theory of their mechanism of action was based on the gate control theory of Melzak and Wall, although many new devices have new mechanisms such as direct medial thalmus pathways, high frequency wide dynamic neuron changes, pseudounipolar *t* cell junction blockade, and closed loop feedback mechanisms^18–20^.

## Methods

A systematic methodology was developed, peer reviewed and published in PROSPERO (CRD42021235384). The systematic methodology included an eligibility criterion and the use of statistical method to evaluated pooled mean differences (MD) along with a 95% confidence intervals (CI)s.

### Aims

The aim of the study was to explore the prevalence of medical device use and their treatment efficacy in non-cancer pain management.

### Search strategy and eligibility criteria

The search strategy used key words of *medical device, pain management devices, chronic pain, lower back pain, back pain, leg pain* and *chronic pelvic pain* using Science direct, PubMed, Web of Science, PROSPERO, MEDLINE, EMBASE, PorQuest and ClinicalTrials.gov.

All clinical trials, epidemiology and mixed methods studies that reported the use of medical devices for non-cancer chronic pain management published between the 1st of January 1990 and the 30th of April 2022 were included. Commentaries, editorials and opinions were excluded along side of all publications published in any other language than English (PRISMA diagram).

### Data extraction

All studies included a population of patients with non-cancer chronic pain that were considered to use medical devices. The data extraction methodology was developed based on a study specific extraction template that included detailed information such as geographical location, age, sex, pain type, interventions and key statistical indicators such as interventions, measures of tool and numeric results. An extraction template specific to the objectives of the study was developed to gather a wider dataset with vital data for statistical analysis. The number of studies was the number of independent RCTs included in analysis, however sub-studies were extracted from the same clinical trials with different duration periods. The results of different stages in one designed study can be regarded as new sub-studies as new rows in data analysis.

Data was extracted by two investigators and any disputes for eligibility was discussed and agreed with the Chief Investigator of the study. All studies included within the analyses were independently reviewed^21^.

### Outcome measures

Outcomes were reported as median, standard deviation (SD), mean and confidence intervals (CI). Mean and standard deviation were extracted as the main outcomes including pre-treatment pain scores at baseline, post-treatment pain scores and pain score changes of each group.

A variety of interventional tools were used to assess the severity and progress of chronic pain. These include visual analogue scale (VAS), 0–10 or 0–100), numeric rating scale (NRS), 0–10), brief pain inventory interference scale (BPI), 0–10), McGill pain questionnaire (MPQ), face pain rating scale (FPRS), oswestry disability index (ODI), supine bridge test (SBT ) , passive straight leg raise (PSLR), pittsburgh sleep quality index (PSQI), beck depression index (BDI), short form of the brief pain inventory (SF-BPI), SF-BPI pain interference with sleep. There are also other multiple tools we did not obtain numerical results in our analysis, such as EQ-5D index, SF-36 (PCS, MCS), Pain Acceptance Questionnaire (CPAQ).

As most widely used tools for assessing pain such as VAS, NRS use a 11-point numeric rating scale from 0 to 10, the following standardisation formula was used to unify all pain scores into the same scale:

$${\text{Scaled Pain Score }} = {\text{ Original Pain Score }} \times { }\frac{10}{{\text{Scale Range}}}$$

As all outcomes of interest were continuous, the calculation based on pain scores was performed by using mean differences (MD) with a 95% confidence interval (CI) to report the effects between the group comparisons.

### Exposures

The exposures of interest were selected based on the key features of medical device interventions used to treat non-cancer chronic pain, including and not limited to a pain condition being the primary or the secondary condition. Neurological and psychological symptoms leading up to the use of medical device within the included population were also considered.

### Statistical analysis plan

A meta-analysis (MA) is a statistical combination of the results of two or more independent studies comparing two interventions. MA produces one estimate of pooling effects from the selected pair of interventions in different 2-arm studies. Studies included in our analysis used a medical device or device-assisted intervention in Experimental group, while placebo or non-active treatment (Sham stimulation) in Control group. To estimate the efficacy of managing non-cancerous chronic pain with the use of medical devices currently available, PMA was conducted based on different subset of studies and clinical assessments. Firstly, PMA was used on studies with the same medical device as the treatment group to see the specific efficacy of each type. However, due to the limited studies of a certain medical device, all included interventions here in Experimental group could be regarded as a whole of “Medical device”, and all extracted studies were included for meta-analysis.

The primary aim of this study was to provide a comprehensive understanding on use of medical devices and their treatment effects. There are multiple outcomes associated Pain level, Motor function, General health status and quality of life, such as Pain intensity, disability index, EQ-5D index and sleep quality index. The difference in efficacy of medical device in a variety of contexts such as age groups, gender groups, study duration and geographical location were also explored.

${\text{I}}^{2}$ and p-value were commonly used to detect statistical heterogeneity. A value of ${\text{I}}^{2}$ larger than 50% with a much smaller p-value indicates strong heterogeneity. Correspondingly, ${\text{I}}^{2}$ less than 50% with a large p-value indicates fairly weak heterogeneity^22^. A random effects model was chosen when there was high heterogeneity, whereas a fixed effects model was used if weak or no heterogeneity was detected^23^. In the presence of high heterogeneity, subgroup analysis was carried out to identify the sources of heterogeneity. To assess the robustness of the pooled results under meta-analysis, sensitivity analysis was applied. Finally, publication bias was evaluated with funnel plots and Egger tests^24^. All results of statistical analyses were produced by R and packages were used to provide outputs in compliance with best practice and reporting guidelines^25^.

### Ethics approval

The study was conducted in accordance with PRISMA Guidelines.

## Results

### Summary of studies included in systematic review

Table 1 presented the characteristics of the 13 studies included in systematic review with 875 participants enrolled. There are a case series study, single-arm repeated measures study, randomized feasibility trial, randomized crossover study and the left 9 randomized controlled trials (RCTs). Chronic low back pain was the most common pain among enrolled patients with 5 studies testing on 222 patients. 7 studies with medical devices used Nerve stimulation among 296 participants and 4 studies used mobile device to assist treatment for 437 participants. One study used Extracorporeal Shock Wave Therapy and one study tested the feasibility of a newly developed activity pacing framework.

**Table 1:** Demonstrates characteristics of the studies included in systematic review.

Study ID	Authors	Publication year	Design	Pain condition	Devices/intervention	Country	Female (%)	Sample size	Average age	Included in MA	Outcomes measures
1	Y. K. Kim et al	2021	Randomized controlled trial	Chronic low back pain	Transcutaneous electrical nerve stimulation (TENS)	Korea	0	32	27.75 ± 3.38	Y	VAS, FPRS, ODI, PSLR, SBT
2	K. B. Chapman et al	2019	A case series	Refractory low back pain	Dorsal root ganglion stimulation (DRG-S)	USA	59	17	57 [34,71]	N	VAS, ODI, EQ-5D index , SF-36 (PCS, MCS)
3	P. Goldstein et al	2020	Randomized controlled trial	Chronic back pain	Cliexa-EASE mobile platform	USA	42		43.23 ± 15.68	N	VAS, BSMs
4	A. A. Nes et al	2017	Randomized controlled trial	Chronic widespread pain	Smartphone-delivered maintenance	Norway	100	48	43 ± 11.12	N	Pain acceptance questionnaire (CPAQ)
5	M. C. Chang et al	2017	Randomized controlled trial	Chronic lumbosacral radicular pain	Bipolar pulsed radiofrequency (PRF) stimulation	Korea	48	50	60.4 ± 16.3	Y	NRS
6	Noam Goldway et al	2018	Randomized, double-blind, placebo-controlled	Fibromyalgia	Amyg-EFP-neurofeedback	Israel	91%	34	35.6 ± 11.82	N	VAS, PSLR
7	P. B. Lee et al	2006	Randomized, double-blind, placebo-controlled study	Chronic lower back pain	pulsed electromagnetic therapy	Korea	47	36	75 ± 5	Y	NRS
8	B. S. Kang et al	2009	Blinded, randomized crossover study	Chronic central pain	Transcranial magnetic stimulation	South korea	45	11	54.82 ± 13.6	Y	NRS
9	D. Antcliff et al	2021	Single-arm, repeated measures study	Chronic pain/fatigue*	Activity pacing framework	UK	86	107	55.25 ± 12.83	N	Multi-tools*
10	M. P. Harvey et al	2017	Randomized, parallel, double-blind, sham-controlled	Chronic Pain	Transcranial direct current stimulation	Canada	79	14	71 ± 7	Y	MPQ, SF-BPI, PSQI
11	A. Çelik et al	2014	Prospective, randomized, placebo-controlled, double-blind study	Chronic low back pain	Extracorporeal shock wave therapy	Turkey	70	30	49.7 ± 8.3	Y	VAS, ODI, BDI
12	G. Forbes et al	2020	Randomised feasibility trial	Chronic pelvic pain	Mindfulness meditation delivered by smartphone app	UK	100	90	35.0 ± 8.6	N	Chronic pain acceptance score (CPAQ)
13	D. D. Odineal et al	2019	Randomized controlled trial	Chronic musculoskeletal pain	Mobile device-assisted	USA	47	190	55.4 ± 11.0	N	MMEs*

Chronic pain/fatigue*: including chronic low back pain, chronic widespread pain, fibromyalgia and chronic fatigue syndrome/myalgiac encephalomyelitis. MMEs*: Analgesic prescribing change (morphine milligram equivalents, MMEs). Multi-tools*: Activity pacing (APQ-28), Pain (numerical rating scale 0–10), Physical/mental fatigue (chalder fatigue questionnaire), Depression (patient health questionnaire-9), Anxiety (generalised anxiety disorder-7), Self-efficacy (pain self-efficacy questionnaire), Avoidance (escape and avoidance subscale of the pain anxiety symptoms scale-20), Physical/mental function (short-form 12), Quality of life (EQ-5D-5L EuroQol five-dimensions, five-levels index score). APQ-28, 28-item activity pacing questionnaire.

#### Meta-analysis

Of the 13 systematically included studies, 6 were used in the meta-analysis with 173 participants. 5 studies (study 1, 5, 7, 10, 11) were all randomized placebo-controlled clinical trials and study 8 was a randomized crossover study and all of them included a primary endpoint of efficacy. Most studies only compared medical devices with placebo or active placebo. These studies tested the use of transcutaneous electrical nerve stimulation and instrument-assisted soft tissue mobilization combined treatment (TICT), Bipolar pulsed radio frequency (PRF), pulsed electromagnetic therapy (PEMT), repetitive transcranial magnetic stimulation (rTMS), transcranial direct current stimulation (tDCS), extracorporeal shockwave therapy (ESWT) among patents with Chronic low back pain (CLBP), Chronic lumbosacral radicular pain (CLRP), Chronic central pain (CCP) and other Chronic pain (CP).

### Meta-analysis for pain reduction in each study

All studies included in this section provided assessment results of pain levels of participants pre- and post- treatment. And multiple tools such as VAS, FPRS, NRS, MPQ, BPI, SF-BPI, were used among included studies.

For study 1, 5, 7, 8, 10, sub-studies were extracted when they provided testing results of different study stages or used different measurement tools for pain level.

**Figure 1:**
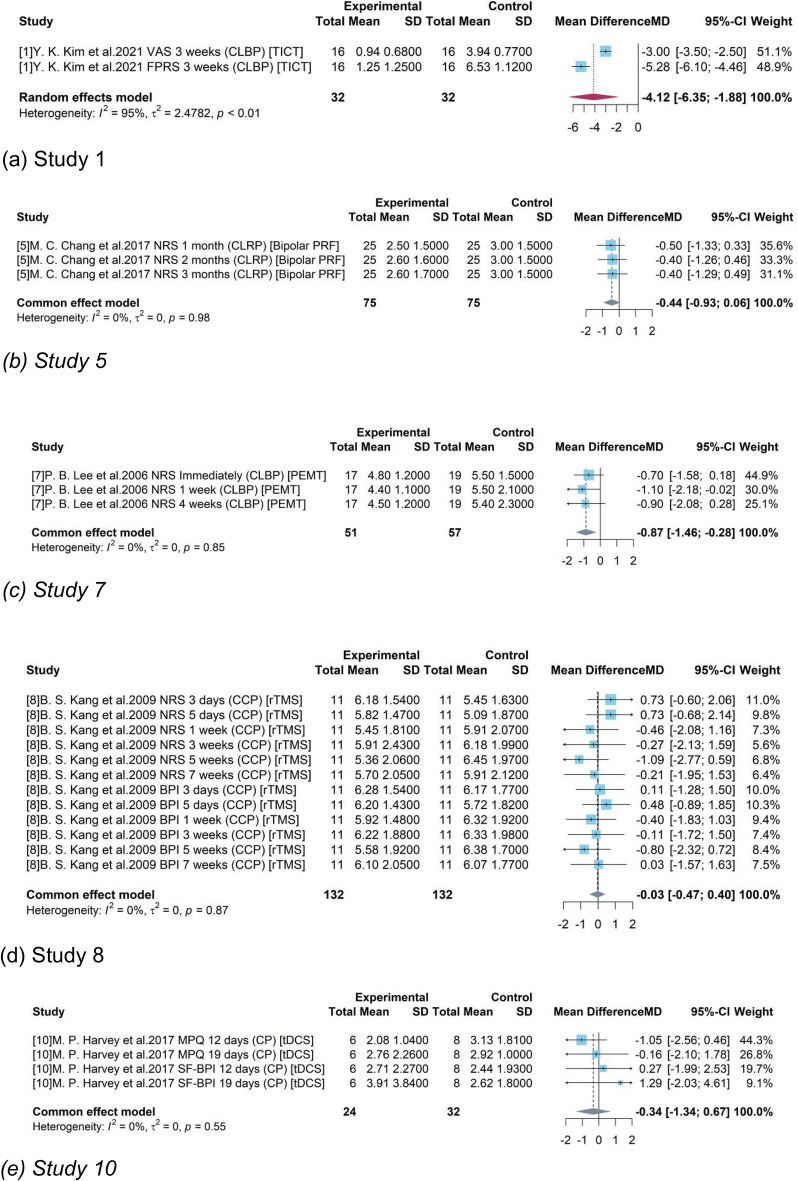
Forest plot for the difference of pain scores in each study.

The pooled results of study 1 and 7 (Fig. 1a, c) were −4.2 (95% CI = [−6.35, −1.88]) and −0.87 (95% CI = [−1.46, −0.28]) respectively. The negative mean difference indicated a pain reduction and the 95% confidence intervals without 0 revealed that TICT and PEMT produced significant treatment on CLBP patients. On the contrary, studies 5, 8 and 10 did not produce a significant results of medical device-related interventions as their pooled estimates had a confidence interval covering 0.

The value of 95% of ${I}^{2}$ (p-value < 0.01) indicated high statistical heterogeneity among the extracted sub-studies based on assessment tools in Fig. 1. Even though VAS and FPRS are both commonly used to assess pain intensity, their assessment results differed a lot due to the gaps between faces representing and a straight line or scale of numbers. It resulted the high heterogeneity between two sub-studies though the numerical results had been standardized.

The weak heterogeneity in other studies with ${I}^{2}$=0 (p-value > 0.05) indicated the feasibility to extract studies based on study duration or assessment tools. As each type of medical device was tested in only one independent RCT, the results from the limited studies are conservative. Therefore, it is needed to call for more RCTs testing on different medical devices with more participants.

### Meta-analysis for pain reduction

Taking the 6 types devices as one group “Medical device” and the placebo-control reference group as the “Control”, we included 6 studies and 25 sub-studies in the following meta-analysis.

**Figure 2:**
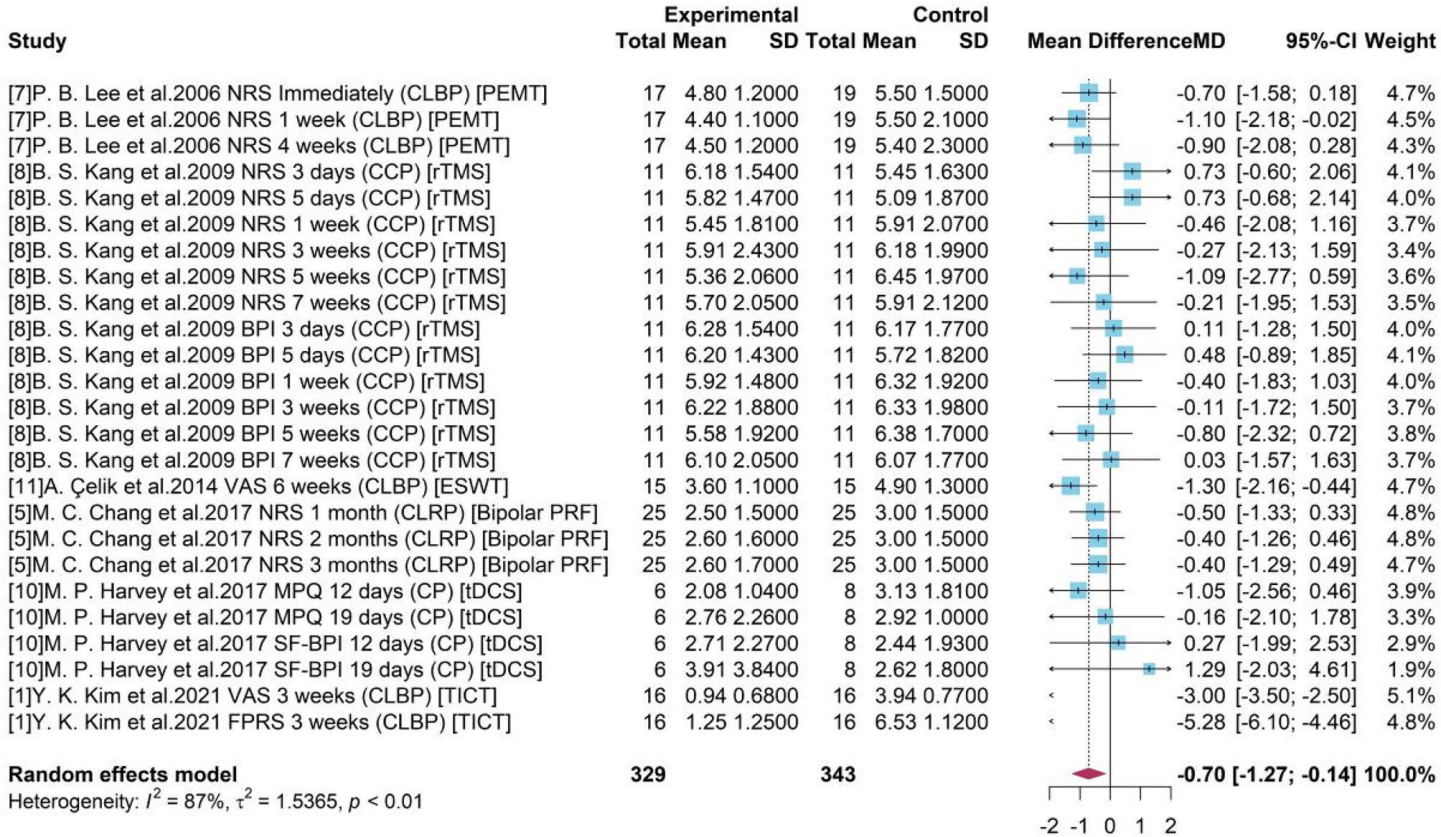
Forest plot for the difference of pain scores with 6 studies.

As presented in Fig. 2, a high heterogeneity was detected with ${I}^{2}$= 87% and p-value < 0.01). The random effects model reported the overall mean difference of medical device compared to control group was −0.70. The 95% confidence interval [−1.27, −0.14] without covering 0 indicated the significance of results. It showed that with the use of medical device-related interventions, patients with chronic pain might have a 0.7 degree of pain reduction under a 0–10 scale.

### Meta-analysis for disability

**Figure 3:**
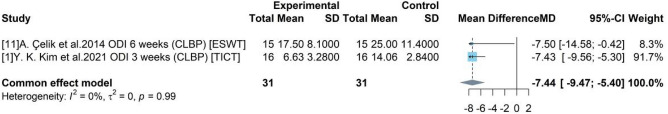
Forest plot for the difference of disability.

As shown in Fig. 3, both of study 1 and 11 reported the ODI, which was used to measure the disability level of daily physical activities (scale range 0–50). All results showed the significant treatment effect of medical devices, including the results in each study and the pooled estimate. With a sample size of 31, the pooled mean difference (MD) of ODI between medical device and control group was −7.44 (95% CI = [−9.47, −5.40]), indicating that medical device treatment could produce a 7.44-degree reduction of disability level in comparison to those using placebo under a 50-score range. There was a weak heterogeneity between these two studies with ${I}^{2}$ = 0% (p-value > 0.05).

### Meta-analysis for sleep quality

**Figure 4:**
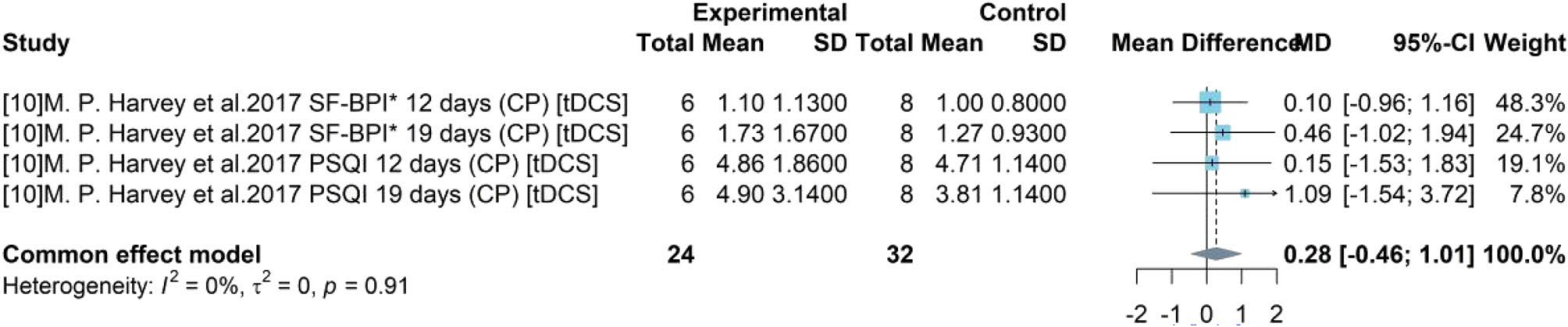
Forest plot for the difference of sleep quality.

Sleep questionnaires (PSQI and SF-BPI) were used in study 10 for assessing sleep quality of participants after treatment. The total score of SF-BPI (pain interference with sleep) PSQI was 30 and 21 respectively and their original numerical results were standardized to facilitate the result explanation. By including 2 stages of this studies and two assessment results, 4 sub-studies were extracted and a sample size of 24 was obtained. As presented in Fig. 4, there was a weak heterogeneity among these sub-studies with ${I}^{2}$ = 0% (p-value > 0.05) and a common effect model was built. The pooled efficacy estimate of device-related intervention transcranial direct current stimulation (tDCS) was 0.28. The 95% CI [−0.46, 1.01] covered 0 and it indicated the insignificance of the pooled efficacy.

### Subgroup analysis for pain level

#### Subgroup analysis with geographical locations

**Figure 5:**
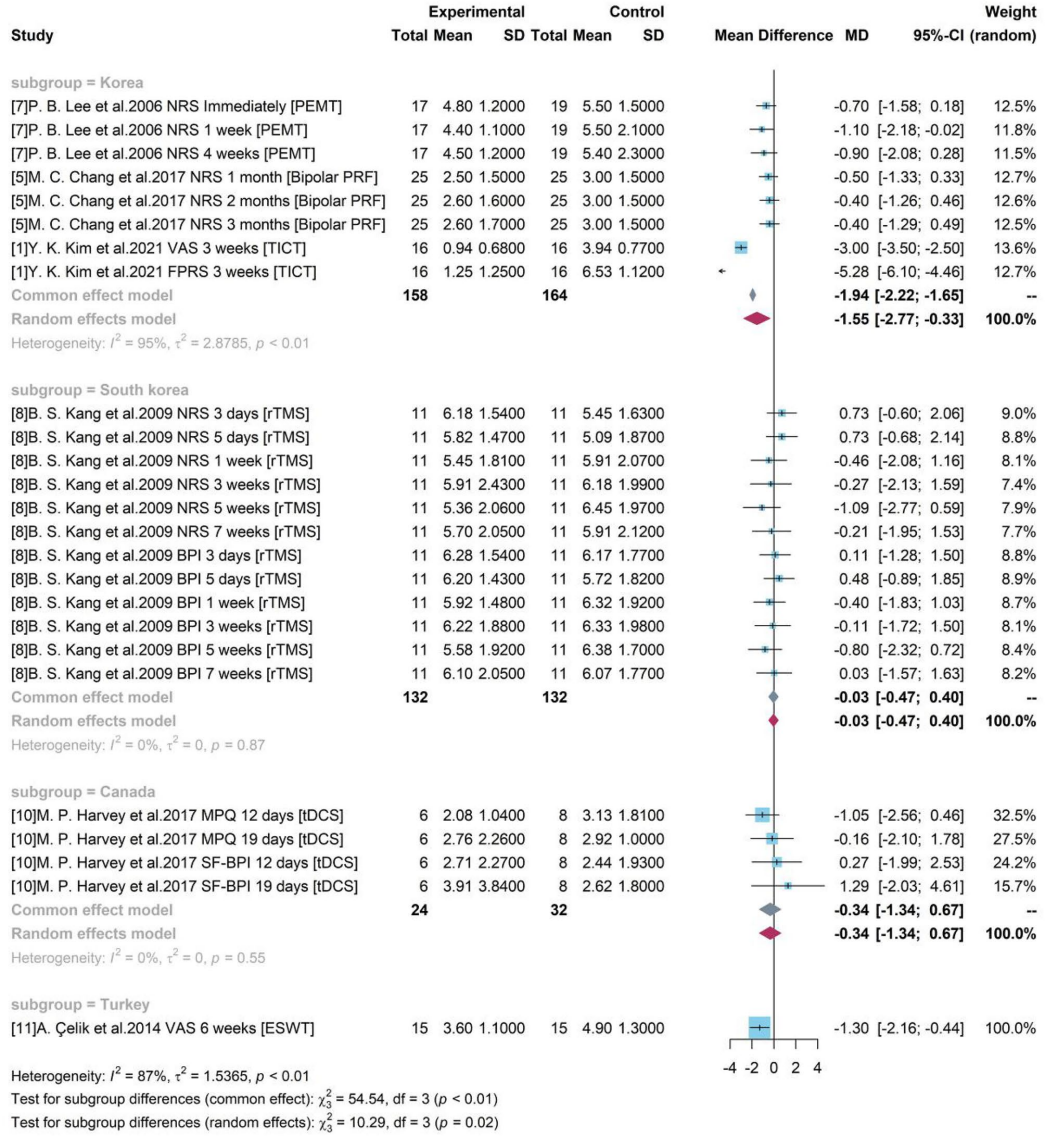
Forest plot for subgroup analysis with geographical locations.

To explore the sources of heterogeneity, a subgroup analysis was conducted using the geographical locations of the studies and demonstrated in a forest plot (Fig. 5). Only study 1, 5, 7 were included in one group “Korea” and other studies did not merge with each other. A statistically significant difference (p-value < 0.05) was identified between group “Korea” and other groups conducted in different countries. The pooled treatment efficacy of studies conducted in Korea was −1.55 (95% CI = [−2.77, −0.33]) and it was significant without covering 0. Figure 5 also showed that heterogeneity was high in group “Korea” (${I}^{2}$ = 95%, p-value < 0.01) and low in group South korea and Canada (${I}^{2}$ = 0%, p-value = 0.87 and 0.55 respectively), indicating that the identified heterogeneity was not geographical location influenced.

#### Subgroup analysis with different age groups

**Figure 6:**
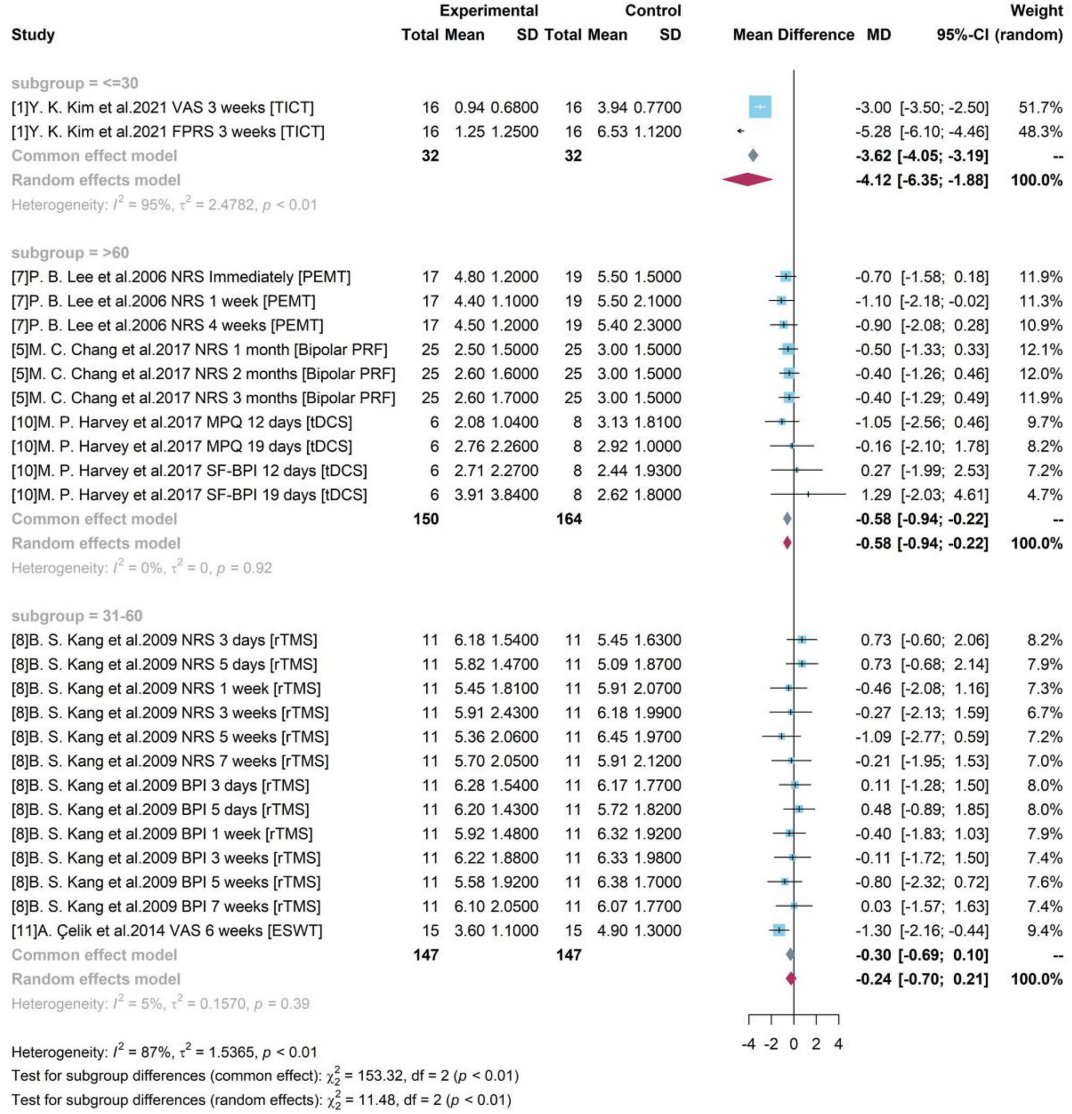
Forest plot for subgroup analysis with different age groups.

As shown in Fig. 6, according to the mean age of enrolled participants, 3 groups were divided as “ <  = 30”, “31–60”, and “ > 60”. Although both of groups “ <  = 30” and “ > 60” produced significant results, the high heterogeneity (${I}^{2}$ = 95%, p-value < 0.01) in group “ <  = 30” caused by two different assessment tools and single source from one study made the result doubtful. For studies with mean age of participants older than 60, the pooled efficacy estimate of medical device-related interventions was −0.58, where PEMT, Bipolar PRF, and tDCS were included. The 95% CI ([−0.94, −0.22]) without covering 0 indicated a significant treatment effect of a 0.58-degree pain reduction. The pooled result of group with participant between 31 and 60 years old was not significant by combining results of study 8 and 11.

#### Subgroup analysis with gender

**Figure 7:**
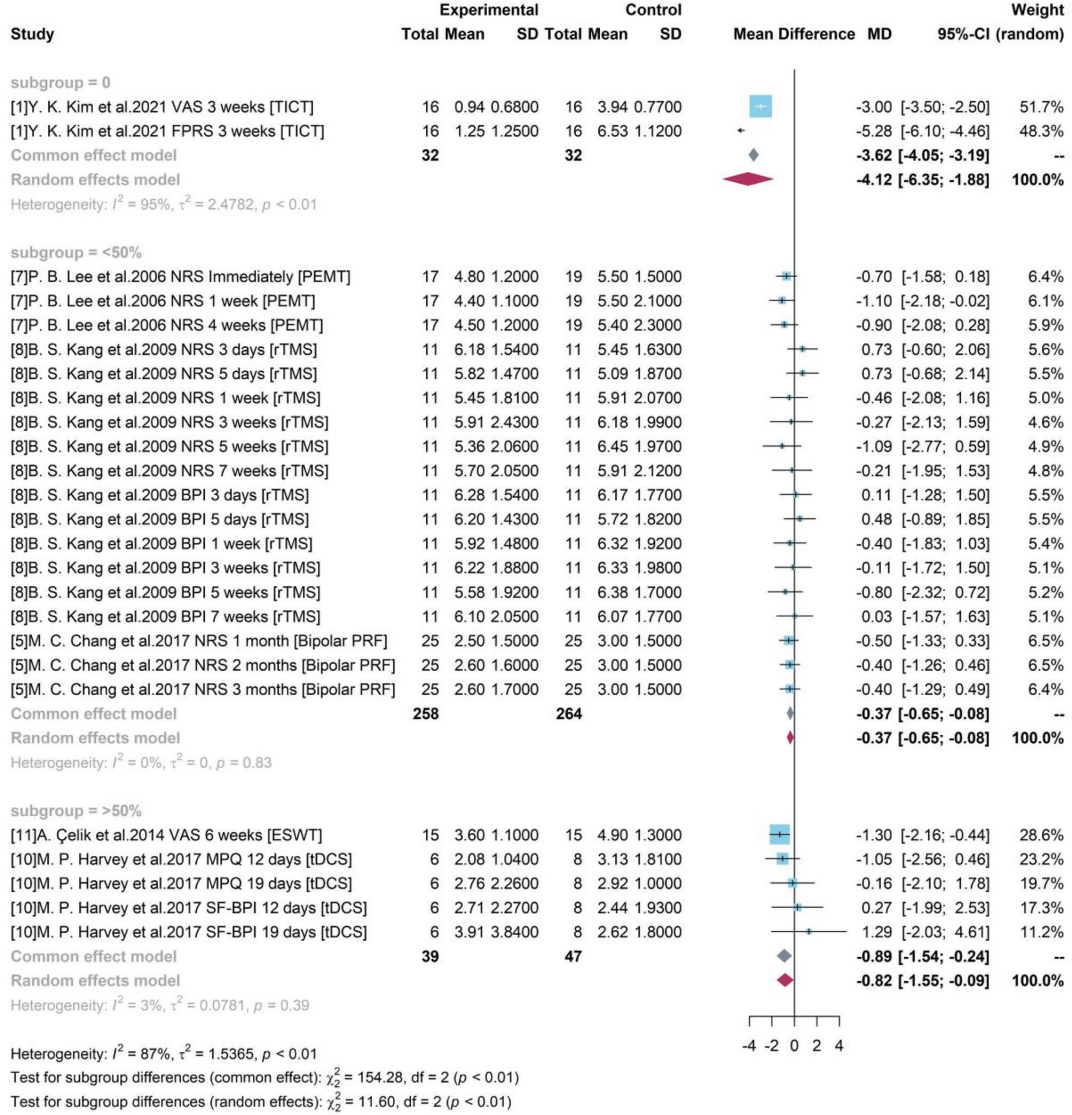
Forest plot for subgroup analysis with different women.

We determined the sample size relevant to women by dividing the 3 groups based on their gender percentage of 0, < 50 and > 50% which indicated these did not have representation from women predominantly. Figure 7 indicates all 3 groups were significant with a zero-free 95% CI.

Study 1 was an all-male study and produced a mean difference of −4.12 between TICT and control group with 95% CI = [−6.35, −1.88]. Studies 5, 7, 8 enrolled women accounting for less than 50% and the pooled efficacy of PEMT, rTMS, Bipolar PRF was −0.37 (95% CI = [−0.65, −0.08]). Studies 10 and 11 enrolled both male and female and female accounts for over 50%. Their pooled result of ESWT and tDCS compared to control group was −0.82 (95% CI = [−1.55, −0.09]).

#### Subgroup analysis with pain types

**Figure 8:**
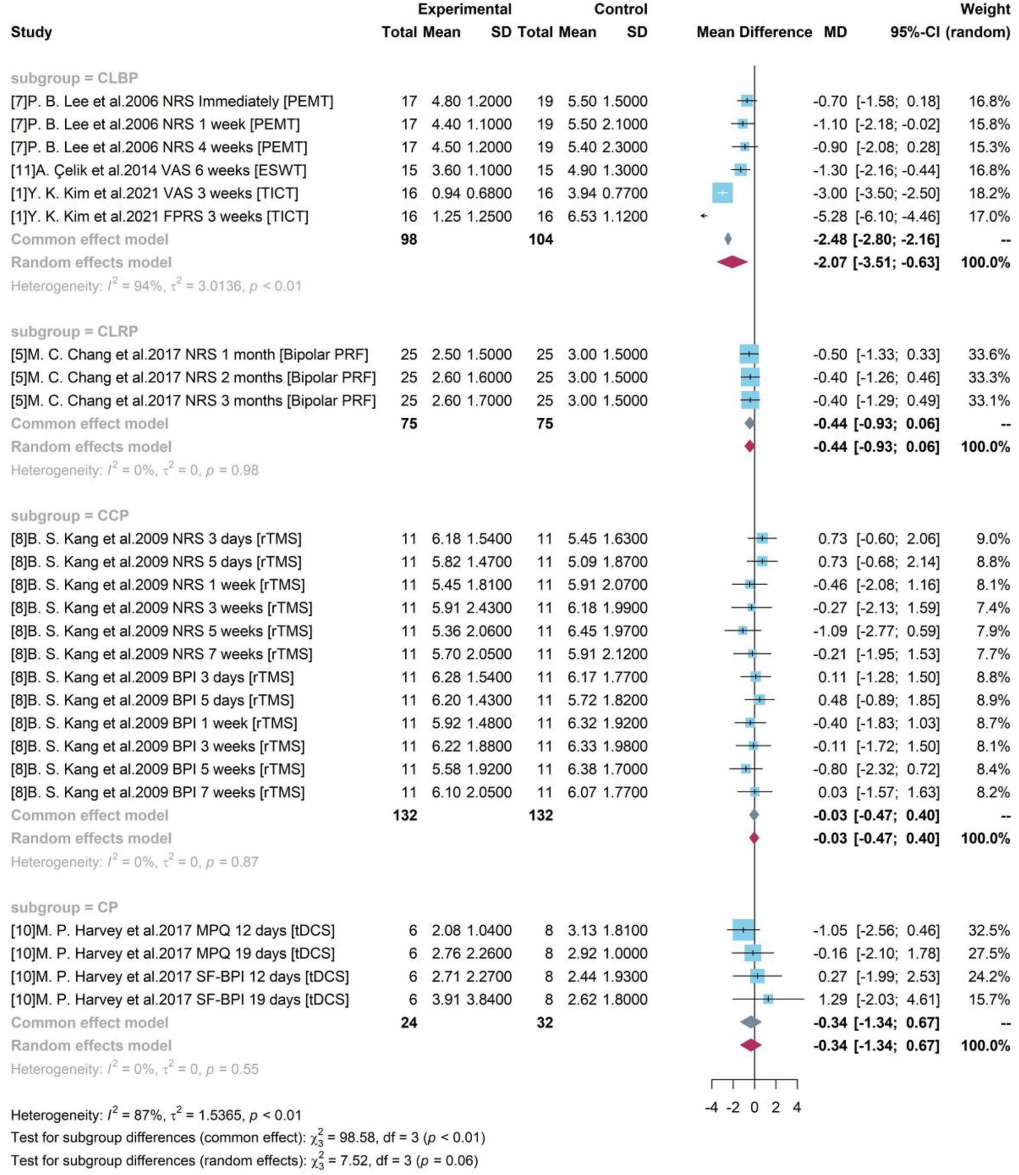
Forest plot for subgroup analysis with different pain types.

Based on different pain types, 4 groups were divided as CLBP, CLRP, CCP and CP. Only studies testing medical device among patients with CLBP produced a significant result. A high heterogeneity was detected with ${I}^{2}$= 94% and p-value < 0.01. Figure 8 shows the random effects model reported the overall mean difference of medical device compared to control group was −2.07. It showed that included medical device-related interventions might produce an averagely 2.07-degree pain reduction on CLBP and a 95% CI ([−3.51, −0.63]) without covering 0 indicated the significance. However, for other groups, only one single study was included for testing the treatment efficacy of medical device on other pain types. And the insignificant results were consistent to the reported results in each study.

#### Subgroup analysis with study duration

**Figure 9:**
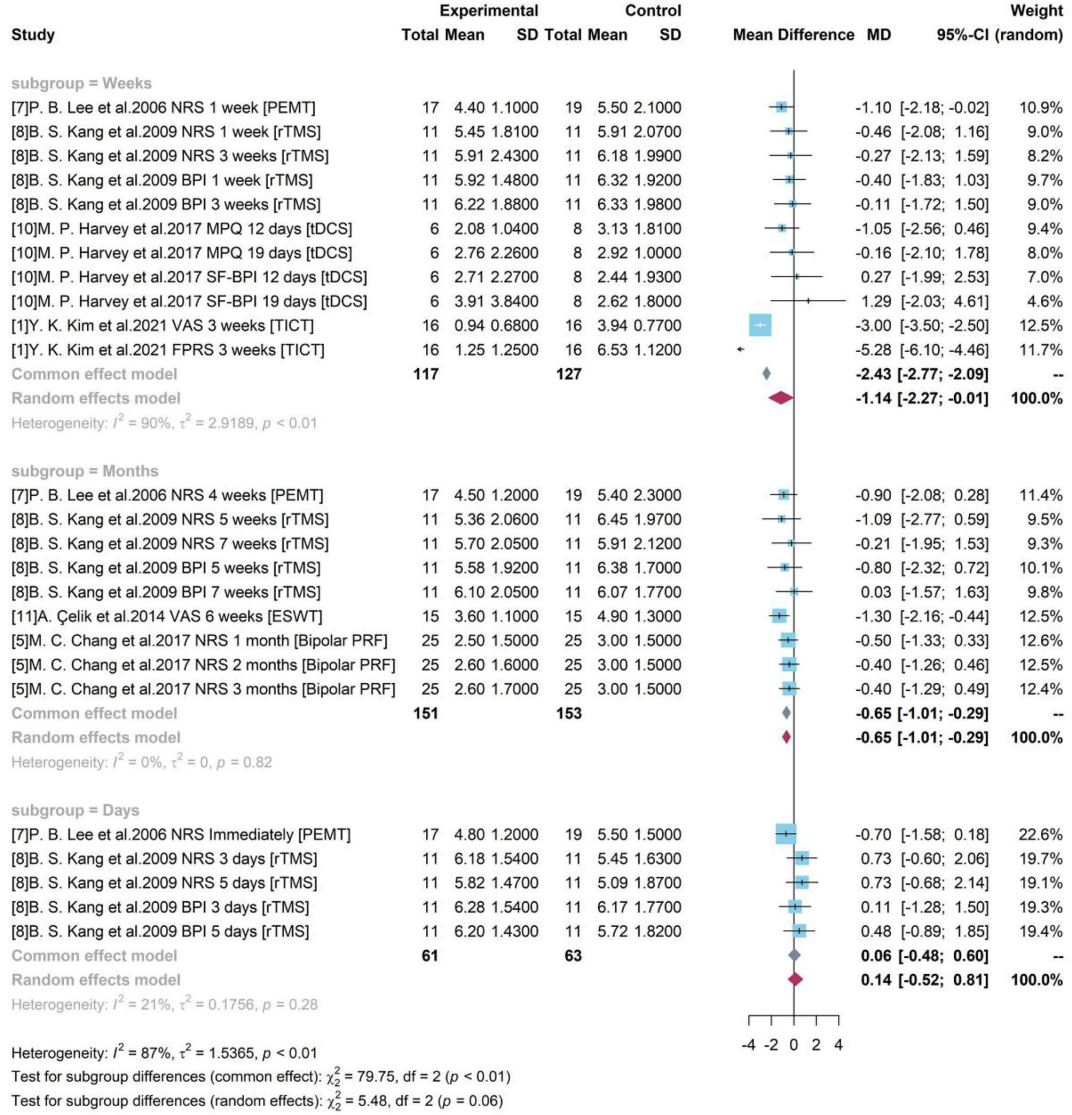
Forest plot for Subgroup analysis with study duration.

Based on the duration of each study, 3 groups were divided as “Days”, “Weeks”, and “Months”. As presented in Fig. 9, an increased heterogeneity (${I}^{2}$= 90% and p-value < 0.01) was detected among studies with testing gap lasting for weeks (including a week) but a decreased heterogeneity in group “Days” and “Months”. It showed that study duration might be one of sources for heterogeneity. The random effects model was used for group “Weeks” and a pooled estimate of device-related treatment effect was −1.14 and the 95% CI [−2.27, −0.01] did not cover 0, showing the significance. The common effect model was used for other two groups. For studies with a gap duration of moths between pre- treatment and post- treatment, a pooled result of −0.65 ([−1.01, −0.29]) was produced. It revealed a pooled efficacy of PEMT, rTMS, ESWT, and Bipolar PRF, the commonly used neural stimulation treatment on chronic pain, was a 0.65-degree pain reduction under a 10-score scale.

### Sensitive analysis

#### Sensitive analysis for all studies used in MA for pain level

**Figure 10:**
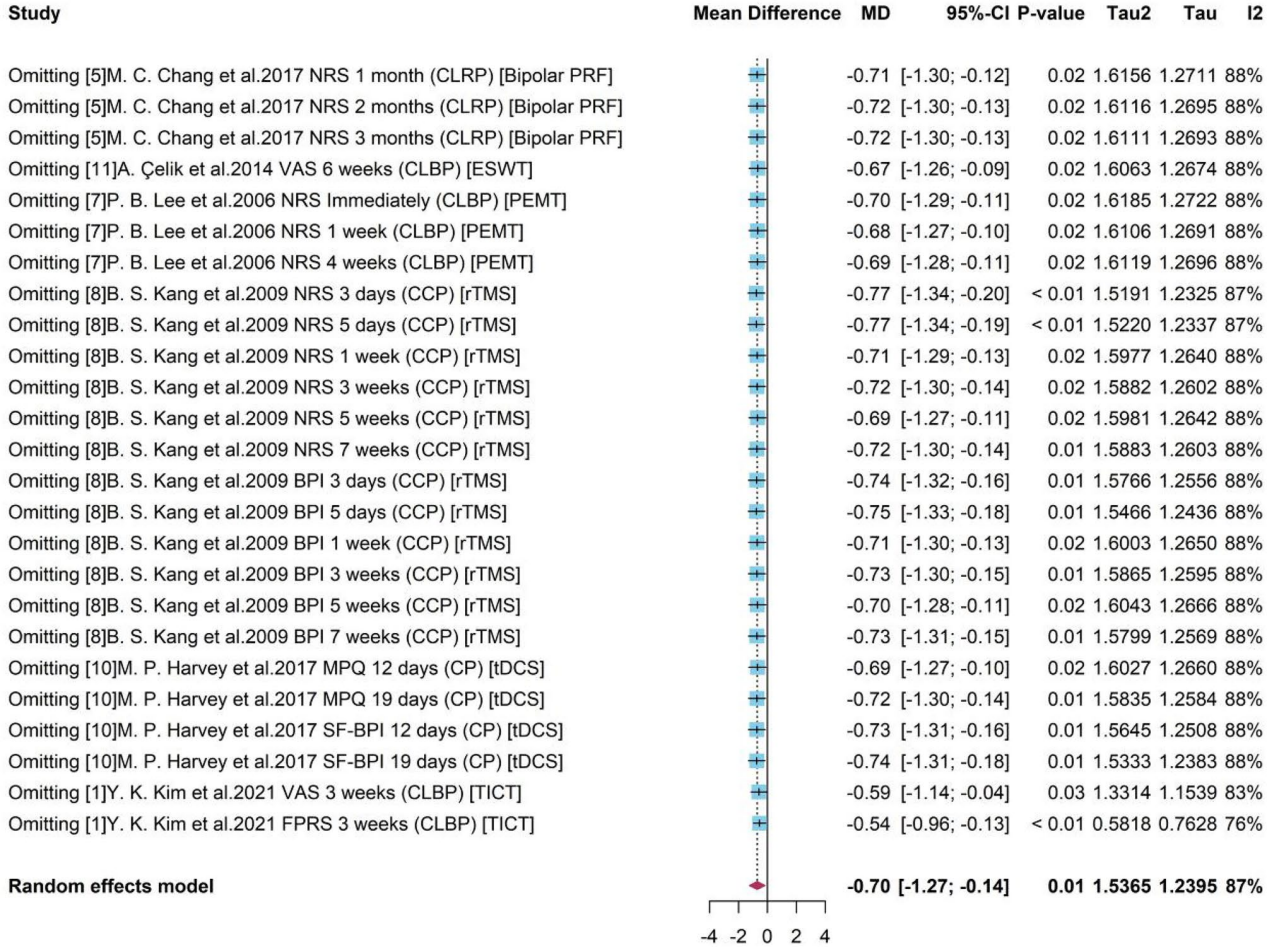
Forest plot for sensitivity analysis with studies in meta-analysis.

To see the robustness of pooled results and detect the possible bias induced by certain studies, Sensitivity Analysis was conducted for studies in MA for pain level. As presented in Fig. 10, on the left were the deleted studies, and on the right were the meta-results of the remaining studies after omitting each study.

It showed that the existence of some studies would influence the pooled result compared to the overall estimation −0.70 with all studies by MA ( Fig. 2). For example, after omitting study 8 the new pooled results like −0.75 and −0.77 would be higher (absolute value) than the overall one. However, without studies 1, an underestimate −0.54 or −0.59 was obtained. It also presented that sub-studies with a duration within a week from study 8 would produce unreliable treatment results, and the absence of them leaded to overestimates of −0.77. The heterogeneity decreased from 87 to 76% after omitting study 1 with assessment FPRS, indicating a potential source of heterogeneity. As a whole, the high heterogeneity (${I}^{2}$= 87%, p-value = 0.01) was stable and a robust treatment effect with negative mean difference and a significant 95% CI was remained.

#### Publication *bias*

**Figure 11:**
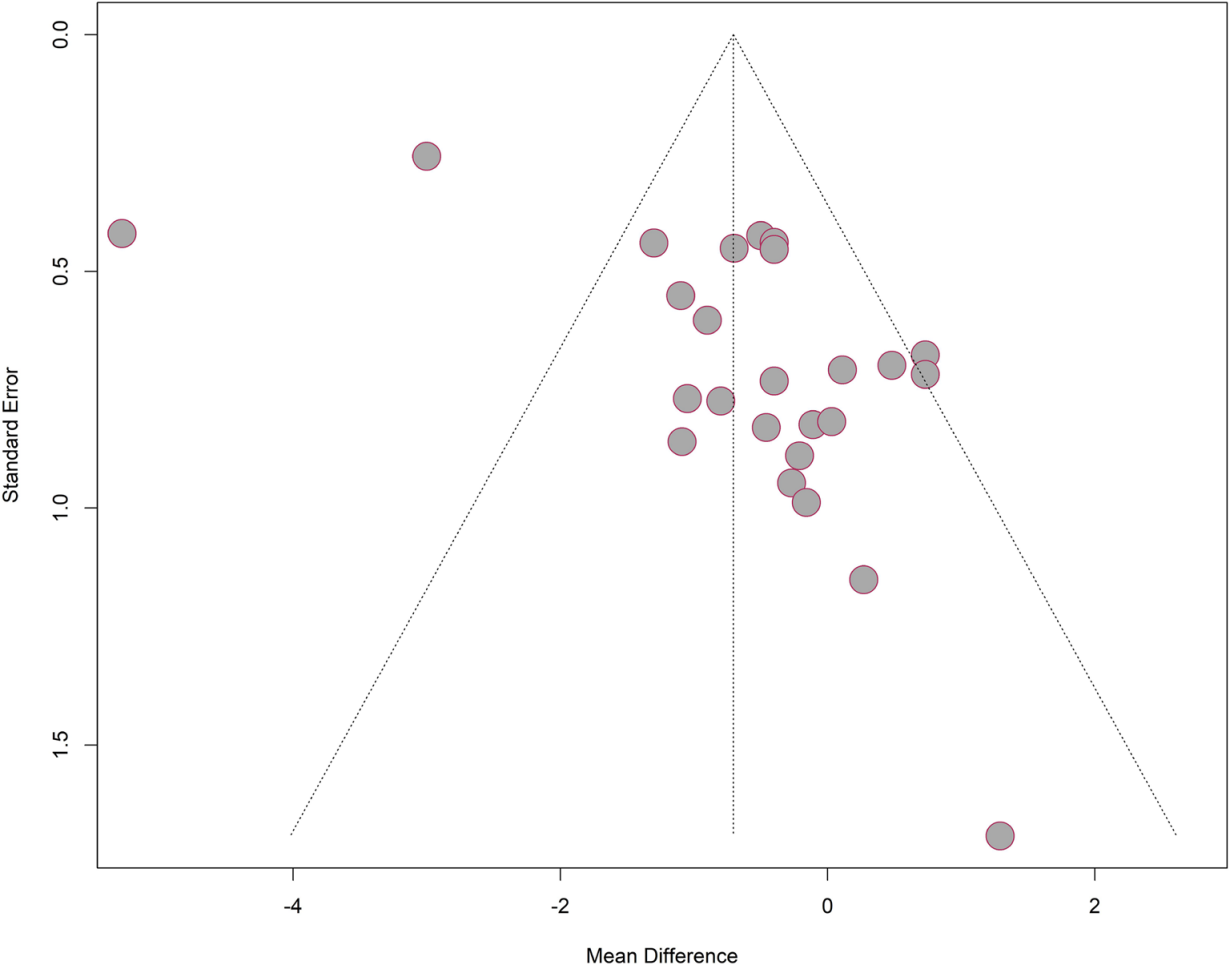
Funnel plot for studies used in meta-analysis.

As presented in Fig. 11, most studies were included under the funnel but the funnel plot for studies used in MA was not symmetric in general. It showed that more studies reported a mean difference closer to 0 but fewer in the left side of the pooled result. As presented in Table 2, the results of Egger test with p value (0.0015) smaller than 0.05 indicated that small-study effects were detected. It can be explained the limited number of studies included in our analysis and most of them had a small sample size. Also, studies with negative results (insignificant treatment effect) reported more stages of study and were extracted into more sub-studies, resulting the more contribution of insignificant results. Therefore, a more reliable and robust result can be produced from more studies testing on medical device with a larger sample size and multiple types of study designs.

**Table 2:** Egger test results for studies used in meta-analysis.

Test result:	t = 3.59, df = 23, p-value = 0.0015			
Sample estimates:	Bias	Se.bias	Intercept	Se.intercept
	4.2590	1.1860	−3.5624	0.6788

## Discussion

The findings of this study indicate that most common medical device clinical trials explore lower back pain with the pooled sample size of 875 patients. Pain reduction was a key outcome in the pooled study sample. Physical therapy is considered as an important facet of strengthening muscles, posture and flexibility. The contextual definition of lower back pain differed across all studies although the reference time was typically defined as pain lasting over 12 weeks. A pathological cause is not always identified although the North American Spine Society defined this as musculoskeletal pain extending from the lowest rib to the gluteal fold that could extend as somatic referred pain into the thigh. Chronic pain localised to the lower back is defined as axial lower back pain and radicular pain is classified as pain that extend to the buttocks and legs. Chronic low back pain can be further classified into post laminectomy pain, also often called failed back surgery syndrome in the literature, and non surgical back pain, which either has no surgically correctable lesions or are not correctable because of patient comorbidities or extensive spinal disease.

The most common pain condition that was treated based on the gathered evidence was lower back pain. All studies did not report demographic data, physical examinations and medical histories. For example, body mass index, weight, smoking status and height was not reported by all studies. These are important aspects to understand both direct and indirect relationships patients may have with pain management. The routine physical examinations performed during the clinical trial eligibility visit is equally useful to understand the neurological origin of the lower back or chronic pelvic pain reported. This is also important to understand if patients in the trials were taking any other medications as the presence of polypharmacy could impact outcomes such as pain intensity and thereby quality of life. The treatments for chronic back pain can be challenging and refractory to a variety of interventions. Spinal cord stimulation (SCS) has shown much promise although the pooled evidence in this study shows immediate relief, most clinical trials did not include longitudinal data. In a clinical setting, SCS is attractive for its ability to improve quality of life, safety, cost and clinical efficacy. This study findings show pain reduction was observed with TICT and PEMT for chronic lower back pain.

The pain disability scores showed significant improvement indicating notable treatment effect. The pooled mean difference of ODI between the medical device and control group was -7.44, indicating a medical device could produce 7.44 degree reduction in disability level compared to a placebo using a 50 score range. The pooled efficacy estimates of the device associated intervention tDCS was found to be insignificant in improving sleep quality. Although the systematically included studies did not indicate if the patients had any pre-existing sleep disorders or any other comorbidity that could lead to poor quality of sleep.

Mobile applications have become a useful application in clinical practice. Whilst some applications are within the medical devices regulations framework, some act as non-clinical support systems for patients with long term conditions. Mobile application based devices are gaining popularity in pain management in migraine, back pain, pelvic pain and fibromyalgia^26^. Of the systematically included studies, 4 studies used mobile applications that are clinician aids to assist with managing pain among 437 patients. Most of the applications identified were able to provide health metrics, symptoms and medical use patterns^27^. Goldstein et al. demonstrated how mobile applications can be used as decision making tools, incorporating machine learning to process large datasets and using this to formulate informed predictions of future pain. The evolution of machine learning and artificial intelligence based systems enabled analysis could emphasise the use of personalised patient management plans in the future. This can be a useful method of long-term management of chronic pain^28^. Despite perceived accessibility and potential for widespread use at minimal cost to healthcare systems it is important to consider the availability of smartphones and the internet in low resource settings^29^.

The subgroup analysis conducted based on gender and pain types showed a disparity between biological gender representation. The subgroup analysis in relation to gender showed studies exploring TICT excluded women and other intervention trials underrepresented women. Whilst this is a common issue noted in clinical trials conducted across most clinical areas, the lack of gender parity is a concern to evaluate clinical efficacy and effectiveness. Equally, physiological differences between genders play a role in reporting pain inference and intensity which is an indicator for patient reported and health reported outcomes that impact cost efficiency^30^. In addition, the lack of gender parity in clinical trials is particularly important to consider in chronic pain management, where women have an increased prevalence in addition to lower pain thresholds, lower pain tolerances and different analgesic sensitivities^6,31^.

Geographical representation is another important facet for understanding the generalisability of the findings. Of the pooled studies, 4 were conducted in Korea and 1 each in Canada and Turkey. The identified heterogeneity was not influenced by geographical location although there may be an indirect link due to differences in clinical practice. An awareness of variations in pain thresholds and disparities in responses to pain treatment amongst different ethnicities remains important although these details were not reported within the identified studies^32–34^.

Better study designs should be considered for future clinical trials. Sample sizes for future clinical trials should reflect the disease and future populations. Some studies had multiple arms and the analysis was performed across the arms to ensure problems arising from the same trial can be examined effectively.

The provenance of evidence described in the findings did not clearly indicate the clinical trial teams comprised of multidisciplinary teams. Official discloses in relation to these studies were also not considered as standard procedure based on the timelines and current research practice guidelines.

It is evident, there is a need for robust clinical trials to better assess medical devices where the findings can be generalisable as indicated by the sensitivity analysis. For example, omitting studies 1 and 8 had a significant effect on the overall mean differences in pain reduction. Whilst, standardizing study designs are not possible, using core outcomes to assess the similar categories of chronic pain may provide better insight to medical device efficacy. The primary outcome measures varied across clinical trials. Pain was also assessed with self-reported measures that could be impacted by factors such as mood, disturbed sleep and medications that may influence the pain scores documented. Further biases may rise from recall period, selective recall, social desirability, or sampling approaches. The pooled sample of studies mostly used descriptive statistics and causal inferences were often not reported. This further purport the self-reported bias could have a difference between the true values versus the self-reported for the same measures.

## Conclusion

The evidence generation to demonstrate efficacy and effectiveness of medical devices in chronic pain management requires extensive changes. Current evidence shows a variety of limitations including restriction to lower back pain when there is a variety of other pain conditions where medical devices are used for such as chronic pelvic pain. Minimally invasiveness in medical devices used in pain management can be a compelling reason for clinicians and patients to continue to use the technique in a cost effective manner. However, to optimally use medical devices in a sustainable manner, robust evidence based practice should be regarded as a key step.

### Supplementary Information

Supplementary Information 1.

Supplementary Information 2.

Supplementary Information 3.

## Data Availability

The authors will consider sharing the dataset gathered upon receipt of reasonable requests. The datasets used and/or analysed during the current study are available from Anish Thillainathan on reasonable request.

## Code availability

The authors will consider sharing the dataset gathered upon receipt of reasonable requests.
