# Supplementary material for: A systematic review and bayesian meta-analysis of medical devices used in chronic pain management
Source: Sci Rep. 2024 Jun 12;14:13549. doi: 10.1038/s41598-024-63499-6 (PMC11169504; doi:10.1038/s41598-024-63499-6)
Supplement: Supplementary file 2 — Supplementary Information 2. [file 41598_2024_63499_MOESM2_ESM.docx]

**Identification of studies via databases and registers**

Records identified from*:

Databases (n =66107)

Records removed *before screening*:

Duplicate records removed (n = 15585)

**Identification**

Records excluded** based on the eligibility criteria

(n = 47376)

Records screened

(n =50585 )

Reports assessed for eligibility

(n =3196)

Reports sought for retrieval

(n =3209)

**Screening**

Systematically included studies (n =13)

Studies included in the meta-analyse (n =6)

**Included**

*From:*  Page MJ, McKenzie JE, Bossuyt PM, Boutron I, Hoffmann TC, Mulrow CD, et al. The PRISMA 2020 statement: an updated guideline for reporting systematic reviews. BMJ 2021;372:n71. doi: 10.1136/bmj.n71

For more information, visit: <http://www.prisma-statement.org/>
