# Supplementary material for: A systematic review and bayesian meta-analysis of medical devices used in chronic pain management
Source: Sci Rep. 2024 Jun 12;14:13549. doi: 10.1038/s41598-024-63499-6 (PMC11169504; doi:10.1038/s41598-024-63499-6)
Supplement: Supplementary file 3 — Supplementary Information 3. [file 41598_2024_63499_MOESM3_ESM.docx]

**Background:**Advancements in evidence synthesis methods have led to increased interest in evidence-based clinical practice. Systematic reviews, which may incorporate meta-analyses to statistically combine treatment effects from various research studies, have become the standard in clinical research. Unlike narrative reviews, systematic reviews reduce selection bias by considering all relevant evidence aligned with the scope of a developed clinical or research question. However, traditional meta-analyses are restricted to comparing treatments directly tested in trials. Network meta-analysis (NMA) addresses this limitation by enabling comparisons of multiple treatments, even if they haven't been directly compared in clinical trials.

**Network Meta-Analysis Methodology:** In recent years, NMAs and multiple treatment comparisons (MTC) of randomized controlled trials (RCTs) have emerged as extensions of pairwise meta-analysis. These methods allow for indirect comparisons of multiple interventions not directly compared in head-to-head studies. NMAs are attractive to clinical researchers and are increasingly used by national health technology assessment agencies and drug regulators to identify the best available interventions. They combine direct and indirect evidence to compare treatments and rank their relative effectiveness. However, NMAs' assumptions are more complex and prone to misinterpretation compared to pairwise meta-analyses, requiring careful consideration.

Traditional meta-analysis is limited to comparing two treatments at a time and cannot assess the indirect effectiveness of treatment comparisons. In contrast, NMAs provide the relative effectiveness of all treatments, even without direct RCT comparisons. The network map represents each treatment with circles (nodes), varying in size based on the number of patients treated in the included study. Lines connecting treatments are weighted by the number of RCTs comparing them, with thicker lines indicating more direct trials. Transitivity is another essential component of NMA, ensuring the similarity between study characteristics to enable indirect effect comparisons with minimal confounding factors. Incoherence can occur when there are discrepancies between direct and indirect estimates, often due to a lack of transitivity. We have compared the interventions in a network but with a Bayesian approach.

**Refinement Protocol:** The extraction and refinement protocol systematically refines the data collected for a systematic review and meta-analysis. This involves finalizing the MeSH terminologies and variables used in the analysis. The protocol follows the Preferred Reporting Items for Systematic Reviews and Meta-Analyses Protocol (PRISMA-P) guidelines and is registered with the International Prospective Register of Systematic Reviews (PROSPERO). The protocol includes specific MeSH terms and keywords for search purposes and outlines data extraction elements such as interventions, comparators, efficacy and effectiveness outcomes, sub-group variables, and any gaps in the core dataset.

**Approach to Evidence Synthesis:**Search results are summarized using a Preferred Reporting Items for Systematic Reviews and Meta-Analyses (PRISMA) study flow diagram. Studies are categorized by design, and their characteristics are reported using frequencies and percentages for categorical variables and means with standard deviations or medians with interquartile ranges for continuous variables. The characteristic table includes all eligible studies systematically included, geographical location, and patient features such as age, gender, and comorbidity status, where applicable.

**Summary of the paper:**

This research paper delves into the complexities of chronic pain management, emphasizing its significant impact on individuals and societies worldwide. Chronic pain affects about 20% of the global population, leading to various challenges such as mobility restrictions, emotional distress, social isolation, and financial burdens. The paper highlights the heterogeneous nature of chronic pain populations, influenced by factors like gender, socioeconomic status, and comorbidities.

It discusses disparities in chronic pain management across different regions, particularly in low-income countries where resources are limited, and access to effective treatments is inadequate. The paper also touches on the impact of global migratory patterns on chronic pain prevalence, with considerations for cultural beliefs and stigmas related to pain.

The focus then shifts towards non-pharmacological techniques in pain management, particularly medical devices. The paper outlines the categories of non-pharmacological treatments, such as neuromodulation devices, and emphasizes the need for robust evidence on their efficacy and effectiveness.

The methodology section describes the systematic approach used in the study, including eligibility criteria, search strategy, data extraction, and outcome measures. It also outlines the statistical analysis plan, including meta-analysis techniques used to evaluate the efficacy of medical devices in chronic pain management.

The results section summarizes the characteristics of the studies included in the systematic review, highlighting the prevalence of lower back pain among enrolled patients. It discusses the findings related to pain reduction and disability scores, as well as the use of mobile applications in pain management.

In the discussion section, the paper reflects on the limitations of the current evidence and the need for robust clinical trials to assess the efficacy of medical devices in chronic pain management. It emphasizes the importance of standardized study designs and outcome measures to better evaluate medical device efficacy across different pain conditions.

Overall, the paper underscores the importance of evidence-based practice in chronic pain management and calls for further research to address the existing limitations and optimize the use of medical devices in pain management.
